# Metformin's Mechanisms in Attenuating Hallmarks of Aging and Age-Related Disease

**DOI:** 10.14336/AD.2021.1213

**Published:** 2022-07-11

**Authors:** Fang-Fang Cheng, Yan-Li Liu, Jang Du, Jun-Tang Lin

**Affiliations:** ^1^College of Life Science and Technology, Xinxiang Medical University, Xinxiang 453003, China.; ^2^Stem Cell and Biotherapy Engineering Research Center of Henan, Xinxiang Medical University, Xinxiang 453003, China.

**Keywords:** metformin, aging hallmarks, antiaging, age-related diseases, healthspan

## Abstract

Aging is a major global challenge, and there is growing demand for new strategies to address the burden of aging. The intensive search for antiaging agents has led to the discovery of a variety of drugs that promote the extension of healthspan and/or life. Metformin is a safe, effective, and globally affordable antihyperglycemic agent that has gained much attention in recent years as a potential antiaging treatment. Metformin has been shown to significantly delay the onset of age-related diseases and increase lifespan in several model organisms. In this paper, we reviewed aging hallmarks and the role of metformin in countering these hallmarks. We examined the beneficial effects of metformin on several age-related diseases and the feasibility of metformin as an agent to extend lifespan and healthspan. Finally, we discussed new research directions to better understand the translational potential of metformin in humans.

It is expected that by 2050, the number of people aged 60 years and older worldwide will reach 2 billion [1]. An increase in the number of elderly individuals is associated with an increase in disability and illness, which impose heavy social and economic burdens on individuals, families and countries. Therefore, there is growing demand for new strategies that address the burden of the aging process. Aging is a progressive, accumulative and natural phenomenon that leads to functional declines in living organisms [2]. Aging is often referred to as a risk factor for age-related diseases, including osteoporosis, type 2 diabetes, atherosclerosis, cardiovascular diseases (CVDs), cancer, liver and kidney failure and immune system dysfunctions, as well as neurodegenerative disorders [3,4].Therefore, aging is sometimes described as the “sum of age-related diseases” [5]. To extend healthspan, efforts are underway to develop antiaging drugs that could slow aging and delay age-related diseases. Multiple clinical trials have suggested the positive effects of metformin on age-related diseases, and the “Targeting Aging with Metformin” (TAME) trial was developed to develop an aging paradigm that can be used to delay aging.

Metformin, which is a Food and Drug Administration (FDA)-approved first-line drug for treating type 2 diabetes, has been used clinically for over 60 years for its effectiveness, safety, and low cost [6]. Numerous studies have shown that metformin treatment enhances insulin sensitivity, induces glycolysis, improves peripheral glucose uptake and suppresses hepatic gluconeogenesis [7,8]. Beyond its antihyperglycemic effects, metformin can exert multiple antiaging effects at the cellular and organism levels [9], which are closely associated with improvements in aging hallmarks, such as inflammation [10], autophagy [11] and cellular senescence [12]. It has been shown that metformin significantly delays the onset of age-associated diseases and effectively reduces the risk of many age-related diseases in humans, including CVD, neurodegenerative disease, cancer, and chronic inflammation [13]. Moreover, the effects of metformin on extending lifespan and healthspan have been demonstrated in several experimental models, including *C. elegans* [14] and mice [15]. Given its excellent effects on age-related diseases and longevity, metformin may be of interest as a potential antiaging agent in humans.

This article aimed to review the role of metformin as a possible antiaging drug. We summarize the hallmarks of aging and the role that metformin may have in countering these hallmarks, highlighting the known and putative mechanisms behind its antiaging properties. In addition, we analyzed the beneficial effects of metformin on several age-related diseases and the feasibility of metformin for extending lifespan and healthspan. Finally, we discuss new research directions to better understand the translational potential of metformin in humans.

## Effects of Metformin on the Hallmarks of Aging

The aging process is a progressive multifactorial phenomenon that exhibits different characteristics at the molecular, cellular, and organismal levels. Deregulated nutrient sensing, DNA damage, the accumulation of reactive oxygen species (ROS), telomere attrition, inflammation, autophagy decline, cellular senescence and stem cell depletion have been identified in aging and play crucial roles during the aging process [16]. Each of the hallmarks of aging is connected to undesirable alterations in cellular, molecular, and physiological functionality, which may represent common features of aging in different organisms. Metformin has important effects on all of these features of aging (Table 1).

**Table 1:** The Hallmarks of Aging and the Effects of Metformin.

Aging Hallmarks	Observations	Effects of Metformin	References
**Deregulated nutrient sensing**	nutrient sensing signaling pathways (mTORC, IIS, AMPK and sirtuins) deregulated during aging; activation of AMPK and sirtuins or suppression of mTOR and IIS can delay aging and promote healthyspan.	decrease insulin and IGF-1 levels and improving insulin sensitivity; restored glucose-sensing pathway by induced changes in the upper small intestinal microbiota; activated AMPK and SIRT1, mediated longevity extension.	[19,26,32-37].
**DNA damage**	DNA damage accrues with age; aberrant DDR and deficient DNA repair are closely associated with aging and premature aging syndromes.	scavenges free radicals, prevents insulin/UV-induced DNA damage and TBI-induced DSBs; stimulates DDR, significantly increases XPC and XRCC1, activates AMPK, promotes DNA damage repair.	[18-24]
**ROS**	high ROS levels contribute to aging onset and progression; oxidant production is increased, and antioxidant enzymes are decreased with aging; the overexpression of catalase increases the lifespan of mice; the accumulation of ROS can reduce lifespan.	decreases ROS by act on mitochondria respiratory complex I and activates AMPK; inhibits TBI-induced oxidative stress by upregulation of SOD, CAT, and GPX1 expression; enhances the activities of antioxidant enzymes in mice treated with CCl_4_, cisplatin, and doxorubicin.	[22,25-32]
**Telomere attrition**	telomeres shorten with cell division; telomerase absence causes age-related pathologies and premature aging phenotypes and is reversed by telomerase overexpression/reactivation.	prevents telomere attrition in the male offspring of mothers with gestational diabetes; increases hTERT expression and activity to delay endothelial cell senescence and protect organism against age-associated atherosclerosis.	[33-36]
**Inflammation**	contributes to most diseases that are typical of old age; proinflammatory cytokines increase with aging; NF-κB is activated in mouse models of progeria and inhibiting NF-κB signaling prevents age-associated dysfunction.	significantly reduces biomarkers of inflammation; decreases the expression of proinflammatory cytokines and enhances anti-inflammatory cytokine levels; suppresses NF-κB; improves macrophage polarization from the proinflammatory M1 to the anti-inflammatory M2 phenotype.	[37-45]
**Autophagy**	aging is associated with reduced autophagic activity; antiaging is related to autophagy induction; autophagy loss in aged MuSCs and CD4^+^T lymphocytes leads to declines in function, and these effects are reversed by autophagy re-establishment; reductions in autophagy regulators accelerate aging.	inhibits nucleus pulposus cell and hPDLC senescence by inducing autophagy; reverses autophagy defectsinCD4^+^T cells from older individuals; enhances mitophagy by upregulating mitophagy-related genes; reduces α-synuclein accumulation by enhancing LC3-II-mediated autophagy in dopaminergic neurons.	[41,46-50]
**Cellular Senescence**	senescent cells accumulate with aging; transplanting senescent cells into young mice drives aging; eliminating senescent cells slows the aging process in progeroid mice and attenuates the aging phenotype.	prevents CSE-induced hBEC senescence; inhibits hyperglycemia-induced endothelial cells senescence; attenuates ceramide-induced and stress-induced cellular senescence; delays senescence in human fibroblasts and MSCs; clears senescent cells and inhibits the expression of senescence-related proteins and the abundance of SASP.	[16,52-59]
**Stem cell depletion**	senescent satellite cells reduce the regeneration of muscle tissues; the number of NSCs and MuSCs decreases and the function and activity of HSCs and ISCs decline with age, which is implicated in tissues degeneration and homeostasis declines, driving aging and age-related diseases.	prevents BMSC senescence and enhances their function in the context of injury; promotes the proliferation and differentiation of NSCs; targets satellite cells to prevent their dysregulation after burn and increases their total numbers; improves mitochondrial function in aged OPCs; restores differentiation and rejuvenation capacities.	[43,47,60-65]

Abbreviations: TBI, total-body irradiation; XPC, xeroderma pigmentosum C; UV, ultraviolet; DSBs, DNA double-strand breaks; DDR, DNA damage response; XRCC1, X-ray repair cross complementing 1; ROS, reactive oxygen species; AMPK, AMP-activated protein kinase; HSCs, haemopoietic stem cells; SOD, Superoxide dismutase; CAT, Catalase; GPX1, glutathione peroxidase; hTERT, human telomerase reverse transcriptase; NF-κB, nuclear factor-kappa B; MuSCs, muscle stem cells; hPDLCs, human periodontal ligament cells; SASP, senescence associated secretory phenotype; CSE, cigarette smoke extract; hBEC, human bronchial epithelial cells ; MSCs, mesenchymal stem cells; ISCs, intestinal stem cells; HSCs, hematopoietic stem cells; NSCs, neural stem cells; BMSCs, Bone marrow stem cells; OPCs, oligodendrocyte precursor cells; T2DM, type II diabetics mellitus; APP, amyloid precursor protein; HFD, high-fat-diet.

## Deregulated nutrient sensing

Aging causes multiple alterations in biological processes; among them, nutrient-sensing signaling pathways have captured much interest because they are deregulated during aging [17]. The mechanistic target of rapamycin complex (mTORC) and insulin and the insulin-like growth factor-1 (IGF-1) signaling (IIS) pathway have prominent aging-modulating effects that act as the major nutrient-sensitive regulator in response to changes in nutrients or growth factors [18]. Evidence has accumulated that inhibition of the mTOR or IIS pathway extends the median and maximal lifespans in yeast [19], *C. elegans*[20], *D. melanogaster* [21], and mice [22-24]. The most efficient measure to extend lifespan, calorie restriction (CR), has been successfully tested in diverse eukaryotic species and has been demonstrated to be associated with reduced IIS and TOR activity [25]. Other nutrient sensors, such as AMP-activated protein kinase (AMPK) and sirtuin signaling, tend to be deregulated with aging. AMPK, a key regulator of cellular energy, is activated in *C. elegans* during nutrient-deprivationor low-energetic conditions and extends the lifespan [26,27], Drosophila [28] and mammals [29] by inhibiting TORC1 activity, activating autophagy, and improving cellular stress resistance. Sirtuins, a class of histone deacetylases involved in lifespan regulation, protect against the aging process by enhancing cellular stress resistance [30]. NAD^+^-dependent deacetylase sirtuin 1 (SIRT1) could also induce autophagy by directly deacetylating AuTophaGy (ATG) proteins, alleviating many age-related diseases and improving the healthspan [31].

Studies have shown that metformin interacts with several known nutrient-sensing pathways associated with aging, with effects resembling those of CR, including postponing aging, extending lifespan and decreasing the incidence and progression of age-associated diseases [32]. Metformin has been shown to decrease insulin and IGF-1 levels and improve insulin sensitivity [33]. Metformin has been the preferred glucose-lowering and insulin-sensitizing pharmacological agent treatment for T2DM. Metformin also induced changes in the upper small intestinal microbiota, increased sodium glucose cotransporter-1 (SGLT1) expression and glucagon-like peptide 1 (GLP-1) secretion, restored the glucose-sensing pathway, and thus reversed glucose-sensing defects induced by a high-fatdiet (HFD) in the upper small intestine [34]. As an established AMPK activator, metformin inhibits complex I of the electron transport chain, leading to an increased ADP/ATP ratio and activated AMPK, which mediates longevity extension in *C. elegans*[35]. SIRT1 can be activated directly by metformin by increasing cellular NAD^+^ levels [36]. Metformin can also exert antiaging effects by inhibiting mTOR in AMPK-dependent and -independent manners [37,38]. Evidence is mounting that metformin can effectively improve nutrient-sensing pathways that postpone aging and decrease the risk for age-associated diseases.

## DNA damage

Among the characteristics of aging, DNA damage has been considered a key factor contributing to aging. DNA damage can be caused by a variety of exogenous or endogenous agents, such as ultraviolet (UV) light, ionizing radiation (IR), genotoxic chemicals, and products of cellular metabolism. Multiple forms of DNA damage can accrue with age in humans, and persistent DNA damage can block transcription and replication, thus promoting cellular senescence and apoptosis, which are cellular phenotypes associated with aging [39]. The DNA repair system and DNA damage responses (DDRs) are essential for preventing and monitoring DNA damage. Emerging evidence suggests that aberrant DDRs and deficient DNA repair are closely associated with aging[40]. Aging and age-related diseases are caused in part by the accumulation of DNA damage, which is probably due to declines in DNA repair capacity, as well as ongoing DNA damage induction [41]. Several premature aging syndromes have underlying genetic DNA repair defects, such as Werner syndrome, in which patients have defects in base excision repair (BER) [40], and Cockayne syndrome and Xeroderma pigmentosum, in which patients have deficiencies in nucleotide excision repair (NER) [42]. Moreover, insufficient DNA repair also plays a role in Alzheimer’s disease (AD) and Parkinson’s disease (PD) [43]. This evidence strongly supports the connection between aging and DNA damage.

Metformin has shown effectiveness in preventing DNA damage and promoting DNA damage repair. Metformin scavenges free radicals, prevents damage to nuclear and mitochondrial DNA, and enhances DNA repair. The pretreatment of cells with metformin followed by culture in the presence of metformin and insulin prevented insulin-induced DNA damage [44]. In a total-body irradiation (TBI) mouse model, metformin significantly inhibited TBI-induced increases in ROS production and DNA double-strand breaks (DSBs) in bone marrow hematopoietic stem cells (HSCs) [45]. Metformin treatment reduced DNA damage and ameliorated spontaneous chromosome breakage in human Fanconi anemia patient-derived cells [46]. Su et al.validated the effectiveness of metformin in reducing the risk of aging by protecting against UV-induced DNA damage [47]. Moreover, metformin may stimulate DNA damage responses, facilitating DNA damage repair and reducing genomic instability. X-ray repair cross complementing 1 (XRCC1) is the major protein in the DNA BER system that is significantly reduced in patients with diabetes, whereas XRCC1 is significantly increased in diabetic patients treated with metformin [48]. Metformin-treated diabetic patients may have higher BER activity than others. Metformin increased the expression of xeroderma pigmentosum C (XPC), a key protein required for NER, and promoted UVB-induced DNA damage repair [49]. In addition, metformin inhibits mitochondrial complex I-mediated oxidative phosphorylation, decreasing cellular ATP levels and thereby activating AMPK, which then activates p53-mediated DNA repair [48]. These results suggest that metformin can prevent DNA damage and promote DNA damage repair, which provides a novel mechanism to explain the antiaging effects of metformin.

## Reactive oxygen species (ROS)

The free radical theory of aging proposes that ROS are a major determinant of aging [50]. ROS accumulation can reduce lifespan by harming cellular organelles and macromolecules [51]. High levels of mitochondrial ROS contribute to aging in genetically modified animals; for example, superoxide dismutase-deficient animals showed typical characteristics of premature aging [52,53]. Increased oxidative stress has been implicated in a variety of age-related pathologies. Several studies have shown that oxidative stress is primarily responsible for the loss of self-renewal and premature exhaustion of HSCs in mice with mutations associated with ataxia telangiectasia; however, prolonged treatment of these mice with an antioxidant could rescue this defect [54]. Under normal circumstances, ROS production and clearance are in a delicate dynamic balance, and the antioxidant system plays an important role in this balance [55]. In aging, oxidant production is increased, while antioxidant enzymes are decreased. Studies have shown that mice overexpressing mitochondrial catalase counteract oxidative damage and live longer [56]. Low ROS levels improve defense mechanisms, which contribute to stress resistance and longevity, while high ROS levels may contribute to aging onset and progression [57]. Therefore, interventions that reduce higher concentrations of ROS under pathological conditions by both scavenging free radicals and enhancing antioxidant defenses are proposed as antiaging strategies.

Metformin decreases ROS production by directly acting on mitochondrial nicotinamide adenine dinucleotide phosphate (NADPH) oxidase and class 1 respiratory chain enzymes and can be considered an antioxidant agent [58]. Metformin interferes with mitochondria and reduces ATP production, leading to the activation of AMPK [59], which has been shown to reduce ROS levels, increase the expression of the antioxidant thioredoxin and induce the antioxidant status of vascular endothelial cells [60]. Studies have also shown that metformin attenuates paraquat-induced elevations in ROS in an AMPK-independent manner [61]. In addition, metformin may also reduce ROS levels by increasing the activities of antioxidant enzymes. The administration of metformin to rats before cisplatin, CCl_4_or doxorubicin treatment restored the activities of GSH, CAT, and SOD [62-64]; metformin inhibited TBI-induced chronic oxidative stress in HSCs by upregulating the expression of the antioxidant enzymes SOD,CAT, and GPX1 [45]. These studies show that metformin reduces ROS levels by inhibiting mitochondrial respiratory complex I or enhancing antioxidant defenses, which may suppress aging onset and progression.

## Telomere attrition

Telomeres act as caps at the ends of eukaryotic chromosomes to protect genetic information from damage caused by the inability of DNA polymerase to completely replicate chromosome ends [65]. Telomere length is an important predictor of species lifespans [66]. Longer telomeres predict less early disease and longer lifespans [67]. Telomerase adds repetitive telomeric DNA sequences at the ends of chromosomes to repair telomeres after cell division [68]. In humans, a lack of telomerase causes a variety of age-related pathologies, including liver disease, pulmonary fibrosis, premature gray hair, and aplastic anemia [69]. In mice, telomerase deficiency is associated with a shorter lifespan and early onset of aging phenotypes, and telomerase overexpression has been demonstrated to increase healthspan and longevity and reverse age-related pathology [70]. These results indicate that the recovery of telomeres is crucial to improving health and even increasing longevity.

Metformin treatment has a beneficial effect against telomere attrition. In mothers with gestational diabetes, metformin treatment prevented telomere attrition in their male offspring [71]. Chronic metformin administration delays endothelial cell senescence by increasing hTERT (telomerase reverse transcriptase, the rate-limiting factor in the formation of functional telomerase) expression and activity, thereby attenuating vascular aging and protecting organisms against age-associated atherosclerosis [72]. HTERT protein and its activity were significantly increased, and all senescence markers were reduced in human aortic endothelial cells (HAECs) that were grown in the presence of metformin compared to untreated cells [72]. However, some studies have reported that treatment with metformin inhibited telomerase activity by decreasing hTERT mRNA levels in endometrial cancer cell lines compared to the control [73]. Further studies are needed to understand the effects of metformin on telomeres in aging and cancer.

## Inflammation

Chronic low-grade inflammation (also termed inflammaging) has been shown to contribute to most diseases that are typical of old age, such as AD, atherosclerosis, cancer, sarcopenia and diabetes [74]. Healthy aging in centenarians correlates with low levels of inflammatory markers [75]. Proinflammatory cytokines typically increase with aging; for example, IL-6 is a hallmark of many chronic inflammatory states in humans and is associated with slower walking speed, impaired muscle strength and sarcopenia [76,77]. In mouse models of progeria, the activation of nuclear factor-kappa B (NF-κB) upregulates the expression of proinflammatory cytokines, while the inhibition of NF-κB prevents age-associated dysfunction [78]. In the fly intestine, preventing activation of proinflammatory NF-κB signaling enhances regenerative homeostasis and extends lifespan [79,80]. These studies suggest that inflammaging is a possible mechanism underlying the aging process [81].

Accumulating evidence suggests that metformin can increase healthspan and lifespan through anti-inflammatory effects [9,82]. Metformin reduces the risk and progression of inflammaging by significantly reducing biomarkers of inflammation. In human studies, metformin was shown to reduce inflammation by decreasing the expression of multiple proinflammatory cytokines (Il-6, IL-1β, TNF-α, MMP-2, MMP-8, AGEs, NF-κB, STAT3, Saa1 and Saa2) and increasing the levels of IL-10 and adiponectin [83,84]. Metformin significantly reduced proinflammatory markers (IL-6, TNF-α, CRP) in the serum of type 2 diabetic patients and suppressed NF-κB levels in the context of diabetes and intestinal inflammation through AMPK-independent and -dependent processes [85,86]. Some findings from experimental models also demonstrated that metformin exerts an anti-inflammatory effect. Metformin administration ameliorates the inflammatory response associated with diabetic nephropathy in type 2 diabetic mice by reducing the increases in NF-κB and serum levels of proinflammatory cytokines (IL-1β and TNF-α) [87]. In HFD-fed rats, metformin decreased the levels of TNF-α, MCP-1, IL-1β and leptin in the intestine (colon), adipose tissue and serum [88]. The anti-inflammatory effect of metformin has also been observed in models of systemic lupus erythematosus (SLE), experimental autoimmune arthritis (EAE), colitis and obesity [89]. A recent paper by Bharath et al. revealed that metformin could reduce proinflammatory cytokines by reducing M1 macrophages and improving macrophage polarization to an anti-inflammatory M2 phenotype [90]. This evidence suggests a promising role of metformin in targeting age-related chronic inflammation via its pleiotropic effects on inflammatory responses.

## Autophagy

Autophagy is a catabolic recycling system that degrades and recycles dysfunctional or unnecessary cellular organelles and molecules to sustain cellular functions. Autophagy and aging have an interconnected relationship. Rubinsztein et al. suggested a causal link between aging and autophagy involving a vicious cycle in which aging causes a decline in autophagy that then exacerbates aging [91]. Studies have also shown that autophagy is essential for longevity and healthspan, and the suppression of autophagy accelerates aging and shortens lifespan [92]. Another type of autophagy that specifically clears damaged mitochondria called mitophagy is also critical for the prevention of aging [93]. Bharath et al. reported that CD4^+^T lymphocytes from elderly individuals exhibit defective mitophagy, which may contribute to aging-associated chronic inflammation, and enhancing mitophagy may ameliorate inflammaging to prolong the health span [90]. The loss of autophagy in aged muscle stem cells (MuSCs) leads to a decline in their function and number, and MuSC aging can be reversed by the re-establishment of autophagy [94]. Animal experiments (yeast, *C. elegans*, Drosophila and mice) demonstrated that the inactivation of or reductions in key autophagy regulators such as Atg1, Atg7, Atg8, Atg11, or Beclin1 accelerated aging and reduced lifespan, while the overexpression of Atg5 or Atg12 delayed aging and extended lifespan [95].

The proautophagic activity of metformin is mainly mediated by the activation of AMPK, which is inactivated in senescent cells and activated by metformin, significantly preventing the development of senescence [95]. Furthermore, metformin alleviated oxidative stress-induced senescence in human periodontal ligament cells (hPDLCs)by inducing autophagy, reversed autophagy defects in CD4^+^ T cells from older subjects and rejuvenated cell functions [90,96]. In a Clk1-mutant PD mouse model, metformin treatment reduced α-synuclein accumulation by enhancing LC3-II-mediated autophagy in dopaminergic neurons and the midbrain [97]. In addition, metformin enhanced mitophagy by upregulating the transcriptional expression of mitophagy-related genes, resulting in the rapid degradation of damaged mitochondria, thereby delaying aging, and improving cardiovascular function in aging [98]. Metformin’s potential in the treatment of neurodegenerative and other aging-related diseases has also been recognized. The neuroprotective effects of metformin mainly involve activating autophagy, which seems to be defective in neurodegenerative disorders [99]. In conclusion, these results indicate that autophagy is a novel therapeutic target for metformin, which may delay the progression of disease, extend lifespan and improve healthspan.

## Cellular senescence

Cellular senescence is considered an aging hallmark based on two factors: (1) senescent cells accumulate in organismal tissues, which parallel age advancement; and (2) senescent cells accelerate the age-related decrease in tissue regeneration [16]. The accumulation of senescent cells in various tissues is thought to be a major factor contributing to aging and age-related diseases [100]. Senescent cells impact neighboring cells and drive systemic aging through the senescence-associated secretory phenotype (SASP) [101]. Transplanting senescent cells into young mice is sufficient to cause physical dysfunction and spread cellular senescence in mouse tissues [102]. Even the functional decline that occurs during normal aging could be slowed by eliminating senescent cells, such as through the use of Bcl-XL inhibitors that can selectively eliminate senescent cells and slow the aging process in the organism [103]. The role of senescent cells in driving aging was also demonstrated by the clearance of senescent cells from progeroid mice carrying a *p16-INK-ATTAC* transgene in which a suicide gene was expressed specifically in p16^INK4a^-positive senescent cells [104]. These results suggested that inhibiting/eliminating senescent cells or suppressing the SASP could reverse aging.

Metformin prevented cigarette smoke extract (CSE)-induced human bronchial epithelial cell senescence by decreasing DEP domain-containing mTOR-interacting protein (DEPTOR) expression [105]. Pretreatment of myoblasts with metformin could attenuate ceramide-induced cellular senescence by limiting increases in p53 and p21 protein levels [106]. Low-dose metformin treatment delayed senescence in human diploid fibroblasts and mesenchymal stem cells by upregulating endoplasmic reticulum-localized glutathione peroxidase 7 (GPx7) [107]. Studies have also shown that metformin attenuates stress-induced senescence in C2C12 myoblasts[106], endothelial cells [108], periodontal ligament cells (PDLCs) [96], nucleus pulposus cells[109] and mouse olfactory ensheathing cells [110]. In addition, metformin could trigger immune-mediated clearance of senescent cells and inhibit the expression of genes coding for multiple inflammatory cytokines during cellular senescence [111]. Metformin treatment could reduce the expression of senescence-related proteins, including p21 and p16, and the abundance of inflammatory cytokines, chemokines and oncogenes that are hallmarks of SASP [112]. The tissue sweeper function of metformin may enhance the clearance of senescent cells and halt the progression of aging.

## Stem cell depletion/exhaustion

Stem cell decline is a prominent, well-established causative factor for aging. The lifelong persistence of stem cells in tissues leads to cellular damage, which may lead to cellular senescence, decreased self-renewal and regenerative capacity, and eventually functional depletion. Moreover, a loss of stem cell numbers and/or function is closely associated with tissue degeneration and homeostasis declines and ultimately drives aging and age-related diseases [113]. During aging, tissues exhibit decreases in the numbers of stem cells, as is commonly seen in skeletal muscle [94] and the nervous system [114]. However, hematopoietic stem cells (HSCs) and intestinal stem cells (ISCs) can maintain their populations or even increase in number with aging but are concomitant with a decline in function and activity [115,116]. The decline in stem cell activity and number with aging results in muscle weakness, hair graying or loss, slower wound healing and decreased immunity [117]. Given the importance of stem cells during the organismal lifetime, the suppression of senescence or restoration of stem cell functions may be a promising therapeutic strategy for geriatric diseases.

There is evidence to suggest that metformin has a beneficial effect on stem cells. Metformin administration decreased stem cell senescence in bone marrow [118] and enhanced stem cell function in the context of injury [86]. Metformin promoted the proliferation of endogenous neural stem cells, increased the total number of neural precursor cells, and improved sensory-motor function after brain injury in mice [119]. Similarly, metformin could target satellite cells to prevent their dysregulation after severe burn injury and increase the proliferation and total number of satellite cells in skeletal muscle[86]. Importantly, metformin restored the function of senescent stem cells. Metformin improved mitochondrial function in aged oligodendrocyte precursor cells (OPCs), contributing to the restoration of differentiation capacity and functional rejuvenation [120]. The effect of metformin on stem cells is complicated, and different types of stem cells may respond differently to metformin, which requires further study.

**Figure 1.**
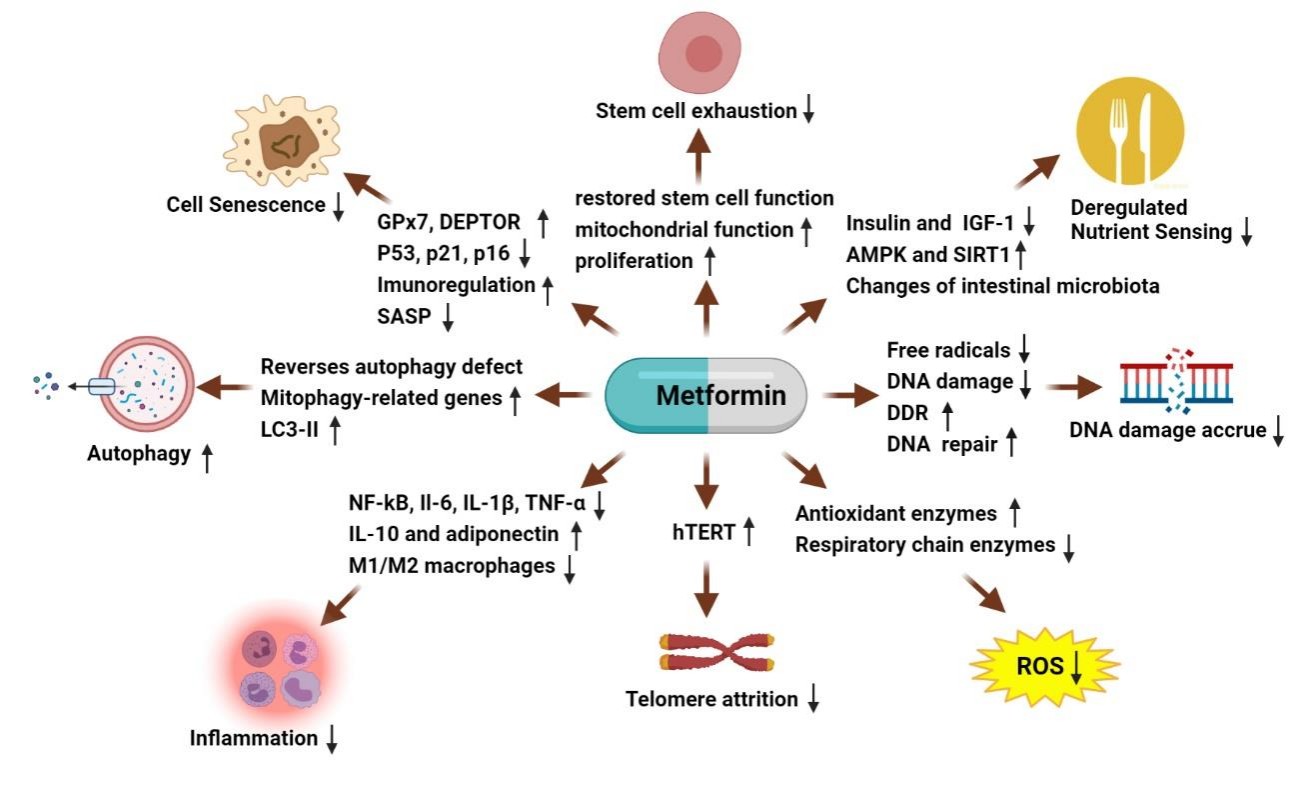
Metformin counters aging hallmarksthrough different pathways.

The aging process is complex, and the interplay of the above hallmarks of aging leads to aging on an organism level. Although the contribution of each of these hallmarks to the progression of biological aging is not fully elucidated, targets that can ameliorate several of these hallmarks can protect against age-related functional declines. Metformin ameliorates the hallmarks of aging through multiple pathways (Fig. 1).Table 1 shows that metformin has a positive impact on the hallmarks of aging. Our understanding of the hallmarks of biological aging and metformin's effects on each hallmark of aging will be helpful for preventing or delaying the progression of aging.

## Effects of metformin on age-related diseases

Aging is an irreversible biological process that is the driving force behind all age-related diseases. Thus, delaying aging slows the onset and development of various age-related diseases and extends lifespan. Metformin exerts antiaging effects by intervening in nutrient sensing, DNA damage, the accumulation of ROS, telomere attrition, inflammation, cellular senescence, stem cell depletion and autophagy and can then be used to prevent aging-related diseases. Over the years, metformin has been shown to exert positive pleiotropic effects on human health. In addition to its ability to improve hyperglycemia, metformin treatment may reduce the risk of many other aging-related diseases, such as cancer, CVD and neurodegeneration [121].

## Metformin and cancer

Metformin initially entered the spotlight as an anticancer agent due to its effects of reducing cancer risk and mortality in patients with diabetes [122]. Since then, several studies have also shown that metformin has utility in cancer prevention and/or treatment [123,124]. The anticancer effects of metformin are realized in several ways. First, metformin reduces insulin and IGF-1 levels [125], both of which can promote tumorigenesis by stimulating the proliferation of epithelial cells. Second, it is widely known that metformin activates AMPK and represses mTOR signaling pathways, leading to cell cycle arrest and apoptosis and inhibiting cancer cell growth and proliferation [126-128]. Third, metformin treatment can impede tumor cell escape from immunosurveillance and enhance the clearance of premalignant/tumor senescent cells (including those with the ability to initiate tumors), thus significantly decreasing the accumulation and malignant/metastatic progression of dysfunctional/ premalignant cells [129]. Fourth, metformin may have a role in inhibiting the self-renewal, proliferation and metastatic ability of cancer stem cells (CSCs)by targeting specific pathways and/or regulatory genes [130]. Metformin can inhibit the proliferation, invasion, and survival and induce apoptosis of ovarian, pancreatic, breast, and colonic CSCs and prevent cancer recurrence and spread [131-134]. Fifth, metformin has been reported to inhibit the activity of the transcription factor NF-κB, leading to reduced secretion of proinflammatory cytokinesthat may have tumorigenic effects [135].

However, some studies have reported inconsistent or conflicting data on metformin's anticancer effects. These studies found that metformin treatment had no association with reduced cancer risk. The reasons for this may be due to diverse analysis methodologies, heterogeneity of studies, different types of cancer or dosage and age at onset of treatment with metformin [136,137]. Although these studies reported inconsistent data, the vast majority of the data support the potential of metformin in decreasing the risk of cancers. We await the results over the coming years to establish a beneficial role of metformin in cancer development.

## Metformin and neurodegenerative diseases

Aging is the greatest risk factor for neurodegenerative disease, and therefore, delayed aging may be an effective way to reduce the occurrence and progression of neurodegenerative disease. The main hallmarks of aging in one way or another are associated with the pathogenesis of neurodegenerative diseases.

Neuroinflammation is considered a major driving force in the progression of neurodegenerative diseases. Substantial evidence suggests that neuroinflammation mediated by microglia plays a pivotal role in PD pathogenesis [138]. Neuroinflammation also contributes to AD pathophysiology, manifested as glial activation and proinflammatory cytokinesecretion, which may lead to neuronal damage [139]. Metformin has certain anti-inflammatory and immunological inhibitory effects. In APP/PS1 mice, metformin exerts its neuroprotective effects by attenuating microglia and astrocyte activation, suppressing inflammatory cytokine secretion and activation and decreasing the expression of proinflammatory factors from activated glia [140]. Additionally, metformin increased microglial phagocytosis capacity and skewed the microglia toward the M2 phenotype [141]. In PD mouse models, metformin reduced the levels of microglia and the proinflammatory cytokines IL-6, TNFα, Il-1β, and iNOS in the substantia nigra pars compacta [97].

Failure of autophagy leads to the accumulation of misfolded/aggregated proteins and damaged mitochondria, which are associated with the pathogenesis and progression of PD and AD [142-144]. The defining pathological hallmarks of AD are the accumulation of neurofibrillary tangles (NFTs, composed of hyperphosphorylated tau protein) and amyloid plaques (composed of extracellular aggregates of amyloid-β) in the brain [145]. SNCA aggregation and mitochondrial abnormalities are key features of PD progression and are associated with impaired degradation mechanisms attributed to deficient autophagy [146]. Several studies have shown that the neuroprotective effect of metformin is associated with autophagic induction. Metformin has been shown to prevent SNCA phosphorylation and aggregation and promote damaged mitochondrial clearance through enhanced mitophagy via AMPK activation [147]. In MTPP/p-induced experimental models of PD, metformin prevented substantia nigra (SN) dopaminergic neuronal death by AMPK-dependent autophagy induction [97]. Similar neuroprotective effects have also been reported in experimental models of AD. In APP/PS1 mice, metformin treatment activates AMPK and reduces the activity of mTOR signaling, thereby facilitating autophagy and promoting lysosomal degradation of Aβ [148], eventually significantly reducing Aβ levels in the brain.

Neuronal death is considered to be the main contributor to cognitive decline in AD [149]. PD is characterized by the progressive loss of dopaminergic neurons in the substantia nigra pars compacta [150]. Recent evidence shows that AMPK activation by metformin contributes to the prevention of substantia nigra dopaminergic neuronal death [97]. In animal models of PD, metformin prevents MPTP-induced dopaminergic neuron degeneration and death via autophagy induction and ROS clearance [97]. In APP/PS1 mice, metformin promoted the repair of damaged neurons and decreased neuronal death and amyloid plaque load [151]. Similarly, cell studies have demonstrated that metformin protects PC12 cells and hippocampal neurons from H_2_O_2_-induced oxidative damage by activating the AMPK pathway and attenuates Cd-induced PC12 cells, SH-SY5Y cells and primary neuron death by suppressing intracellular abnormal ROS accumulation [152].

These results suggest that metformin can exert a significant neuroprotective role by targeting the hallmarks of aging. Metformin may be a promising therapeutic strategy against neurodegenerative diseases.

## Metformin and cardiovascular disease

CVD is the leading cause of death in the elderly. The rise in the incidence of age-related CVDs poses a serious threat to human health and longevity. Aging often serves as an independent risk factor for CVD, leading to progressive structural and functional deterioration of the heart and vasculature mainly manifested as endothelial defects, cardiac and vascular remodeling and loss of vascular compliance [153], increasing the susceptibility of individuals to atherosclerosis, stroke, coronary artery disease, hypertension, heart failure, and atrial fibrillation.

The potential cardiovascular protective effects of metformin are also an active area of research, which reduces the incidence and all-cause mortality of cardiovascular events [154]. Metformin may reduce the risk of CVD by reducing cholesterol levels, blood pressure, body weight, and the progression of atherosclerosis [155]. Animal studies have shown that metformin delays vascular aging and protects against age-associated atherosclerosis in ApoE^-/-^ mice [72]. Metformin has strong protective effects against CVD by improving lipometabolism, reducing the level of LDL cholesterol, and attenuating cardiomyocyte contractile defects by activating AMPK [156]. Metformin similarly reduces the risk of atherosclerosis in diabetic and nondiabetic individuals by improving endothelial cell function and reducing the inflammatory response, which participates in the formation of atherosclerotic thrombi [157]. Metformin exerts a direct vascular anti-inflammatory effect by inhibiting NF-kB, which activates multiple pro-inflammatory factors, including the pro-atherogenic cytokines IL-6 and IL-8 [158]. Metformin alleviates hyperglycemia-induced endothelial impairment and suppresses myocardial apoptosis and inflammation by downregulating autophagy [159,160]. However, other studies report paradoxical data about the regulation of autophagy by metformin. These studies found that metformin provides cardioprotection in δ-sarcoglycan deficiency-induced dilated cardiomyopathy by enhancing autophagy [161]. Therefore, it is still necessary to clarify additional mechanisms of the cardiovascular protective effects mediated by autophagy. The overall evidence from both clinical trials and experimental animal studies supports the benefit of metformin in CVD.

## Effects of metformin on healthspan and lifespan

**Figure 2.**
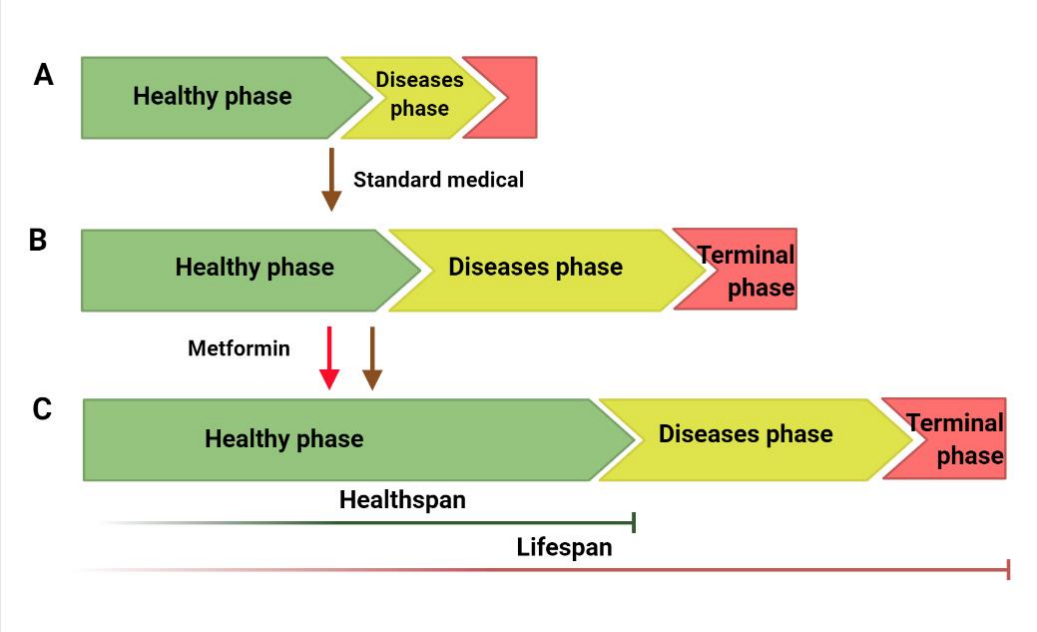
**Effects of metformin on healthspan and lifespan**. Standard medicineextends the “unhealthy” phase of life (from A to B). Diet with metformin at thepreddisease stage extends lifespan and healthspan (From A to C). Healthy phase, before overt diseases become apparent; disease phase, characterized by symptoms of overt diseases; terminal phase, characterized by organ damage, failure and loss of functions.

Worldwide, the proportion of older people is rapidly increasing. Modern medicine has proven that aging is the main risk factor for adult diseases [162]. Therefore, the health issues of older individuals are increasingly important. Standard medical treatments are initiated when overt diseases are diagnosed, which extends the “unhealthy” phase of life by preventing death from these diseases (From Fig. 2A to 2B). This strategy led us to the awkward position of longevity with poor health. A significant number of individuals will rely on drugs through the latter half of life. Therefore, extending the healthspan has been gaining attention in recent years. To date, aging is unavoidable, but the outlook on delaying the aging process and disease onset to extend healthspan is optimistic. Given the effects of metformin in attenuating the hallmarks of aging and preventing age-related disease, metformin has shown much promise as a potential antiaging drug. Diet with metformin at the stage of prediseases not only extends lifespan but also the ‘healthspan’, which is the period in which people remain disease-free and vigorous [13,163] (From Fig. 2B to 2C).

A number of studies support the role of metformin in extending healthspan and lifespan in different animal models, such as worms and rodents [164]. In rodents, metformin treatment reduces chronic inflammation and the accumulation of oxidative damage, induces autophagy, delays the onset of carcinoma, inhibits the growth of tumors, and improves cardiovascular health, all of which can contribute to delaying aging and extending healthspan [165,166]. In nematodes, metformin treatment extended the mean lifespan, reduced the accumulation of lipofuscin and prolonged the healthspan through AMPK [35]. In addition, the effects of metformin on lifespan and health have been demonstrated in humans. T2DM patients taking metformin had significantly lower all-cause mortality than nondiabetic individuals. A meta-analysis by Campbell et al. included 53 studies showing that T2DM patients taking metformin have less CVD and cancer than other diabetic patients and lower mortality than nondiabetic patients as well as other diabetic patients receiving nonmetformin therapies, such as insulin and sulfonylurea [167]. A recent randomized comparative study showed that combined with metformin, both liraglutide and sitagliptin, reduced body weight, intrahepatic lipids, and visceral adipose tissue in addition to improving glycemic control and thus significantly reduced the risks of nonalcoholic fatty liver disease and cardiovascular events in patients with T2DM [168]. Metformin administration has also been shown to be beneficial in improving lipid metabolism, including decreasing plasma triglyceride (TG) and low-density lipoprotein (LDL) cholesterol levels, and reduces mortality and morbidity in diabetic patients with heart failure and atherothrombosis compared to sulfonylurea [169]. However, metformin’s effects are not consistent. First, different initiation times of metformin treatment have different effects on lifespan. Diet with metformin that begins early in life leads to a healthier and longer lifespan in mice, but initiation at older ages shows reduced efficacy [15]. The effect of metformin on lifespan and health was attenuated with increased age at initiation. Second, different doses of metformin can produce different results. Metformin exhibited a typical dose response in some animal studies. For example, treatment with a low dose of metformin starting at middle age extended the healthspan and lifespan of mice and nematodes, while a high dose caused toxic effects and significantly shortened the lifespan [15]. Third, the effect of metformin varied according to sex and species. Metformin extended the mean lifespan of male silkworms without a significant effect on females [170] and prolonged the lifespan of female mice but not males[171]. Moreover, not all studies have shown similar effects of metformin on lifespan or healthspan. Some studies have shown that metformin treatment at a low dose did not increase lifespan in flies, and there was a significant decrease in survival in response to a high dose [172]. In conclusion, the effects of metformin on healthspan and lifespan vary depending on the species and sex of the animals, as well as dose and initiation time.

The mechanisms by which metformin extends healthspan and lifespan have not yet been fully elucidated. One well-accepted mechanism is that metformin can mimic the effects of dietary restriction by stimulating AMPK to reduce energy-consuming processes [107]. Recent studies also indicated that metformin indirectly extends lifespan by altering the gut microbiome, especially by changing microbial folate and methionine metabolism [173,174]. To date, there has been no evidence of such effects in humans. Further work is needed to determine the mechanism by which metformin prolongs healthspan and lifespan and whether metformin leads to improvements in healthspan and lifespan in humans.

## Conclusions and perspectives

The present review demonstrates that metformin ameliorates some of the hallmarks of aging, delays or prevents age-associated diseases, and extends lifespan and healthspan. Considering these promising findings and the safety and long-term use of metformin in humans, metformin is currently considered a hopeful candidate drug against aging and age-associated diseases. However, the application of metformin as an antiaging drug in nondiabetic individuals is still at a very early stage, and several questions have to be clarified. 1) We need to develop a standard score for aging. This score could be used by researchers to assess the effect of interventions that modulate human aging. 2) Metformin’s antiaging efficacy in nondiabetic patients needs to be verified. The ongoing metformin trials are almost all based on observations of potential benefits in diabetic populations. It remains unknown whether nondiabetic individuals taking metformin will live longer and healthier without side effects. 3)The doses and initiation time of metformin treatment for antiaging in humans need to be determined. 4) The mechanisms underlying metformin's protective effects against aging remain to be explained. It is unclear whether metformin protects against aging by targeting multiple aging pathways downstream or by direct effects on multiple aging regulators. 5) It is necessary to highlight the potential side effects of long-term metformin use, such as lactic acidosis and vitamin B12 deficiency, although these side effects are very rare. Therefore, further studies are still required to firmly establish a beneficial role of metformin in healthy individuals. Two large clinical trials, ‘TAME’ and ‘Metformin in Longevity Study (MILES)’, have been initiated in the United States and aim to assess the usefulness of metformin in nondiabetic patients to determine whether metformin can delay aging and improve human health span. Metformin can be used to delay aging and extend human healthspan and lifespan, and the future is promising.
